# Analysis of Insertion Torque of Orthodontic Mini-Implants Depending on the System and the Morphological Substrate

**DOI:** 10.3390/jfb16080291

**Published:** 2025-08-13

**Authors:** Tamara Rahela Ioana, Filip George Boeru, Ioana Mitruț, Anne-Marie Rauten, Mahmoud Elsaafin, Mihaela Ionescu, Ionela Elisabeta Staicu, Horia Octavian Manolea

**Affiliations:** 1 Department of Orthodontics, Faculty of Dental Medicine, University of Medicine and Pharmacy of Craiova, 200349 Craiova, Romania; tamigmg@yahoo.com (T.R.I.); anne.rauten@umfcv.ro (A.-M.R.); elisabeta.staicu@umfcv.ro (I.E.S.); 2 Private Practice “Embrace”, 200618 Craiova, Romania; filipboeru@gmail.com; 3 Department of Dental Technology, Faculty of Dental Medicine, University of Medicine and Pharmacy of Craiova, 200349 Craiova, Romania; 4 Department of Orthodontics, Faculty of Dentistry, George Emil Palade University of Medicine and Pharmacy of Targu Mures, 540142 Targu Mures, Romania; nabil.elsaafinmahmoud@umfst.ro; 5 Department of Medical Informatics and Biostatistics, Faculty of Dental Medicine, University of Medicine and Pharmacy of Craiova, 200349 Craiova, Romania; mihaela.ionescu@umfcv.ro; 6 Department of Dental Materials, Faculty of Dental Medicine, University of Medicine and Pharmacy of Craiova, 200349 Craiova, Romania; horia.manolea@umfcv.ro

**Keywords:** orthodontic mini-implants, insertion torque, bone quality

## Abstract

Orthodontic mini-implants are well-known anchorage devices and stand out as a particularly effective tool for ensuring maximum anchorage without relying on patient compliance. Therefore, it is necessary to understand what levels of torque strains remain in the physiological limits and can guarantee the stability of these mini-implants. The aim of this study was to investigate and measure the initial and final torque values of orthodontic mini-implants when placed perpendicular to the maxillary and mandibular bone surfaces. In our study, orthodontic mini-implants from different companies were inserted perpendicularly using different insertion torques on the plate of both maxillary and mandibular bones from pig specimens. The torque values were then analyzed. The results of this study highlight the need for continued research to analyze the ideal insertion torque of different types of mini-implants depending on the insertion area, in order to achieve clinical success of mini-implants.

## 1. Introduction

Recent advancements in anchorage control methods have highlighted the growing importance of temporary anchorage devices (TADs), commonly known as orthodontic mini-implants. These devices have proven to be particularly effective in providing maximum anchorage without depending on patient cooperation. Their straightforward insertion into bone tissue enables the direct application of orthodontic forces to the skeletal structure, offering a stable bone interface and reliable anchorage within physiological limits [1]. Therefore, it is necessary to understand what levels of torque strains remain in the physiological limits and can guarantee the stability of these mini-implants. Orthodontic mini-implants have been introduced as promising solutions to this problem, but their results are not always consistent. These devices can loosen, become mobile, or even migrate [2].

The stability of orthodontic mini-implants is influenced by two main factors: (1) patient-specific variables, such as age, sex, oral hygiene, cortical bone thickness, proximity to dental roots, and whether the implant is placed in the maxilla or mandible and (2) properties of the mini-screws themselves—namely their design, diameter, and length—as well as surgical considerations like insertion torque, angulation, and the selected placement site [1].

Orthodontic mini-implants have become one of the most commonly used bone anchorage devices, valued for their ease of use, patient comfort, and ability to support immediate loading. However, this same immediate loading can also pose a risk to their stability, potentially compromising their performance. Very good stability is required, due to the immediate loading on the mini-implants, which varies depending on the patient’s bone, the mini-implant design and clinical technical factors [3].

The stability of orthodontic mini-implants (TADs) is influenced by several still unclear factors. Excessive insertion torque may lead to bone necrosis and implant failure. Insertion torque, measured in Newton centimeters (Ncm), reflects the friction between the screw and bone and serves as an indicator of mechanical stability. Stability is classified as primary, i.e., mechanical stability achieved at insertion, and secondary, which develops over time through bone remodeling at the implant interface [2].

Achieving primary stability requires a certain level of maximum insertion torque. However, animal studies have linked high torque values and overtightening with cortical bone fractures. Orthodontists are interested in whether specific torque ranges correlate with higher success rates, and if so, what factors influence these torque values. Identifying a safe and effective torque range remains a key goal in optimizing implant outcomes. Maximum insertion torque values in the range of 5 to 10 Ncm have been presented as the gold standard in several clinical articles [2].

The primary stability of mini-implants depends on several factors, including their length, diameter, number of flutes, thread configuration, the density and thickness of cortical bone, the insertion method, and the anatomical location where they are placed. In contrast, secondary stability develops progressively as a result of bone healing processes, such as regeneration and remodeling [4]. Experimental studies on animals often assess the biological stability of mini-implants by considering both the loading time and screw design.

Applying excessive insertion torque during placement may jeopardize the success of mini-implants, as it can damage the surrounding cortical bone and even lead to necrosis. The maximum insertion torque (MIT), measured in Newton centimeters, represents the highest torque value recorded during the placement procedure [5]. It reflects the frictional resistance between the implant threads and the cortical bone, and is a widely used parameter for evaluating the mechanical stability of mini-implants.

For optimal clinical performance, temporary anchorage devices (TADs) should be manufactured from materials with mechanical properties capable of providing sufficient stability to withstand immediate loading, without undergoing long-term structural changes. Titanium alloy (Ti-6Al-4V) and austenitic stainless steel (AISI 316L) are the most common materials used for TADs. However, in clinical scenarios involving higher cortical bone density and thickness—such as in extra-alveolar sites—selecting TADs with superior mechanical strength may reduce the risk of fracture [6].

The aim of this study was to investigate and measure the initial and final torque values of orthodontic mini-implants when placed perpendicular to the maxillary and mandibular bone surfaces.

## 2. Materials and Methods

The study was conducted in accordance with the Declaration of Helsinki and approved by the Ethics Committee of the University of Medicine and Pharmacy of Craiova (No. 61/18.04.2022, approved date 18 April 2022).

Pigs have 44 teeth in their permanent dentition, 11 on each hemiarch. They present three incisors, one canine, four premolars and three molars. The analysis of a hemi-arch highlights an edentulous area between the canines and the first premolar. In our study, the posterior area was used starting from the first premolar. This area presents anatomical bone characteristics similar to those of humans.

To avoid animal sacrifice for ethical reasons, pigs were used as experimental subjects, using bone segments of the oral cavity from animals already sacrificed for food purposes. Therefore, for this study we selected pig bone samples obtained from a local butcher shop and we tested 3 types of self-drilling orthodontic mini-implants made of titanium alloy.

**Figure 1:**
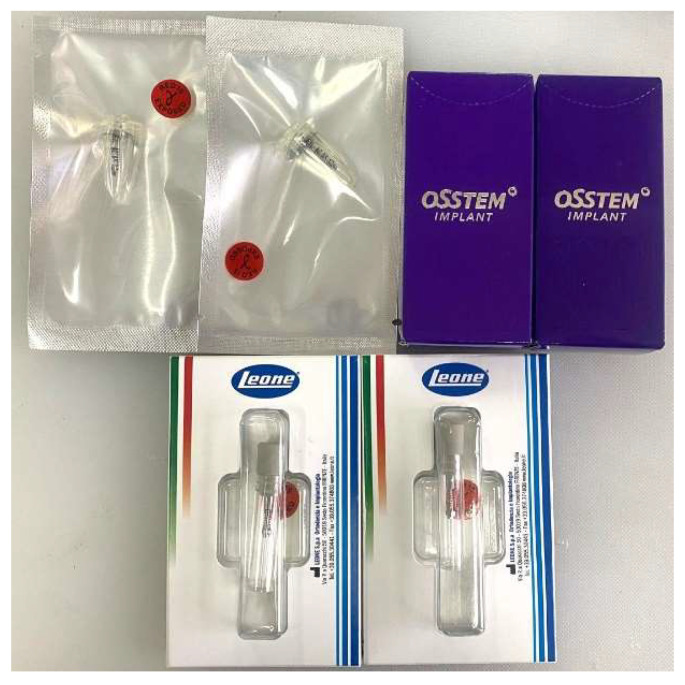
Dental implants from the brands Jeil-Medical, Osstem and Leone.

For this study, we used 12 TADs made of aluminum–vanadium titanium alloy from 3 systems (Figure 1) produced by the following companies:

- Dual Top Anchor System (Jeil Medical Corporation, Seoul, Republic of Korea);
- OrthAnchor System (Osstem, Eschborn, Germany);
- Leone System (Leone orthodontic products, Sesto Fiorentino, Firenze, Italy).

In the present research, twelve mini-implants made of Ti6Al4V alloy were used. The dimensions of the mini-implants were the following: for Jeil Medical, a 2 mm diameter with a total length of 10 mm; for Osstem, a 1.8 mm diameter with a 10 mm length; for Leone, a 2 mm diameter with a 9 mm length.

In this study, we used 16 pig bone samples, 8 hemimaxillary bone samples and 8 hemimandibular bone samples.

**Figure 2:**
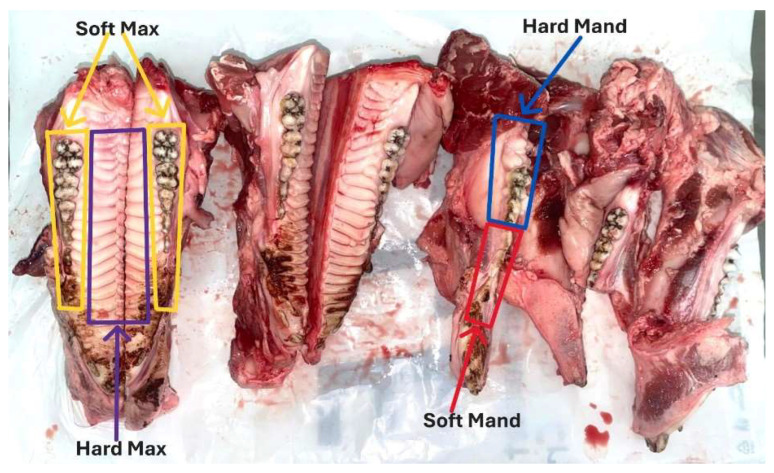
Localization of the 4 defined bone morphological zones in the maxilla and mandible of the pig.

In each bone sample, we inserted six (6) mini-implants, 3 mini-implants in the SOFT area and 3 mini-implants in the HARD area (Figure 2), with 1 mini-implant each from the three systems: Jeil-Medical, Osstem and Leone.

Each mini-implants was inserted twice, first in a bone sample and second in another bone sample, which determined the name of each reading with a letter and two digits, thus encoding the type of implant (J—for Jeil mini-implants, O—for Osstem mini implants, L—for Leone mini-implants), with the first digit being the number of the implant inserted from 1 to 4, and the second digit the number of the first or second insertion.

All the mini-implants were placed using a surgical handpiece by the same operator (T.R.I.), using the speed of 25 rpm, and the maximum insertion torque value of each implant was recorded in Ncm as displayed by the surgical device screen. The mini-implants were inserted by using the protocol suggested by the producers, using the self-drilling technique, where the mini-implant drills itself into the bone without a pilot hole.

The detailed geometrical characteristics of these screws are provided in Table 1. The mini-implants were bought from the Romanian market, and there was no involvement of the manufacturers.

To facilitate the analysis of the insertion areas of the orthodontic mini-implants, the bone areas were divided into 4 insertion zones. Starting from the premise that areas with lower bone density require lower insertion torques, we divided the bone substrate from the used bone segments into two types of areas for each arch. Thus, areas that were considered from an anatomical perspective to have a lower bone density were named SOFT AREAS and areas that were considered from an anatomical perspective to have a higher bone density were named as HARD areas, as detailed below:

1. Soft Mand—represents the mandibular interdental area of the premolars and premolar edentulous area;
2. Hard Mand—represents the mandibular interdental area of the molars and the retromolar area;
3. Soft Max—represents the maxillary interdental area of the molars, premolars and premolar edentulous area;
4. Hard Max—represents the maxillary area at the level of the palatine vault.

**Figure 3:**
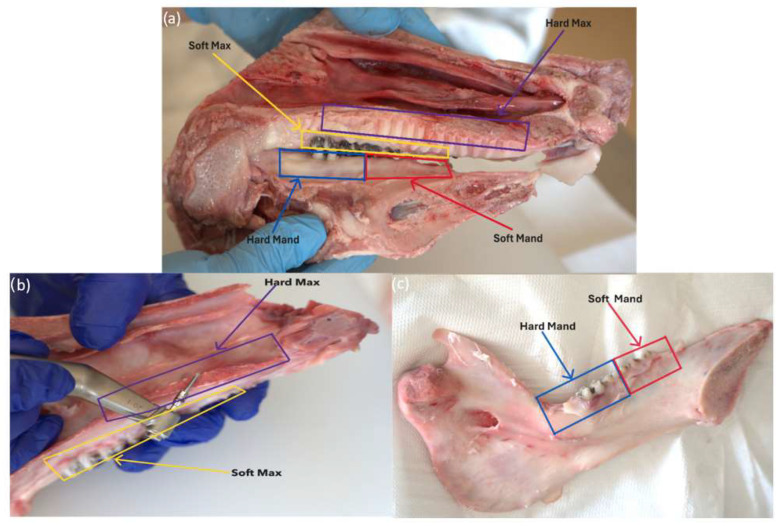
(**a**) Overview morphological areas of the maxilla and mandible; (**b**) maxillary; (**c**) mandible.

In each of these morphological areas (Figure 3), three mini-implants from each system were inserted and 12 study lots were obtained.

The orthodontic mini-implants were placed using several insertion torques: 5 Ncm, 10 Ncm, 15 Ncm, 20 Ncm, 25 Ncm, 30 Ncm, 35 Ncm and 40 Ncm.

**Figure 4:**
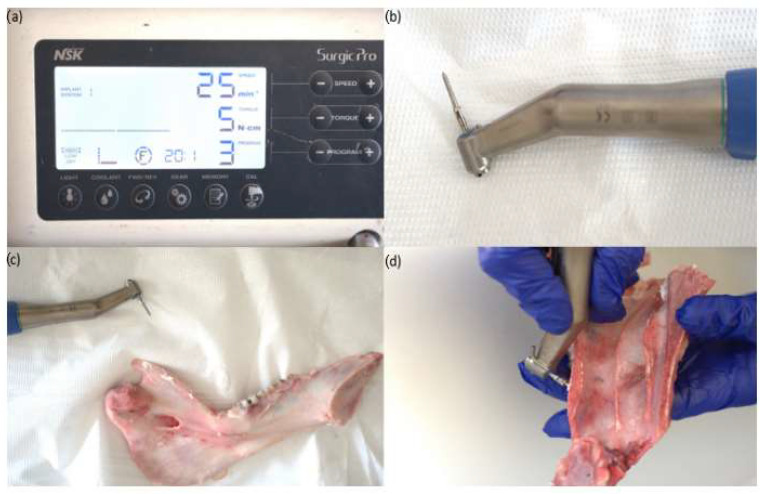
(**a**) The surgical device (**b**) The handpiece used for the TAD insertion (**c**) The handpiece with the attached TAD for a mandible sample (**d**) The handpiece with the attached TAD for the maxillary sample.

The insertion site was carefully selected and the screws were inserted perpendicularly on the plate of both the maxillary and mandibular bones. Before each measurement, the torque indicator was calibrated accordingly (Figure 4).

**Figure 5:**
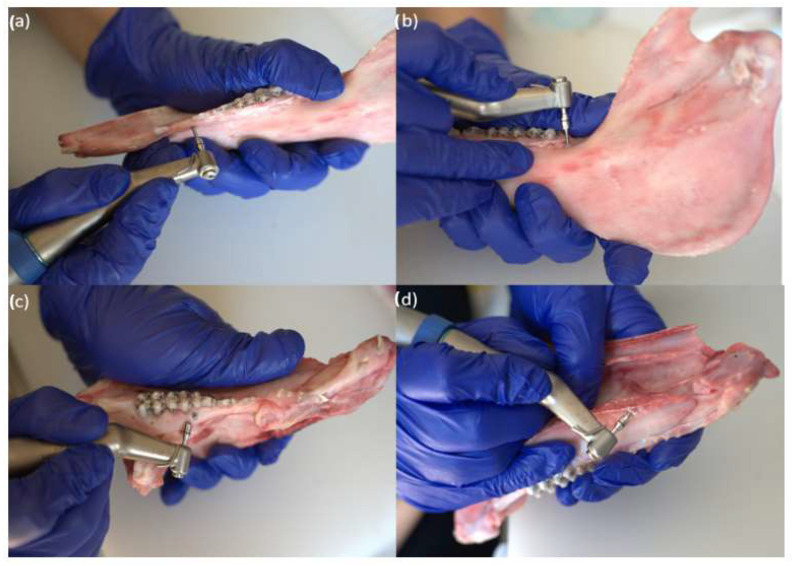
(**a**) Soft Mand; (**b**) Hard Mand; (**c**) Soft Max; (**d**) Hard Max.

For screw insertion, the authors used the NSK iSD900 (NSK-Nakanishi, Kanuma, Tochigi, Japan) handpiece (Figure 5).

### Statistical Analysis

All recorded torque values were entered into a table using Excel software (Redmond, Washington, DC, USA). The graphs were automatically generated using the same software, employing the Box and Whisker chart option, which was created directly from the acquired data table. SPSS (Statistical Package for Social Sciences) software, version 26 (SPSS Inc., Armonk, NY, USA) was used to compute further descriptive statistics. For each series of torque values, the Shapiro–Wilk test was used to evaluate the normality, given the small number of samples from the study. Thus, based on the results obtained, continuous data series were described as median values, completed by confidence intervals, minimum, maximum and variance parameters. Comparisons between various groups were performed using the Mann–Whitney U test, as well as Kruskal–Wallis H test, which was followed by pairwise comparisons performed using Dunn’s procedure, with a Bonferroni correction for multiple comparisons, when statistically significant differences were identified. Results were considered significant based on the threshold of statistical significance set to 0.05.

## 3. Results

The torque values obtained for the four defined morphological zones show us the differences between the soft and hard zones in both the maxilla and the mandible, proving their judicious choice (Table 2).

**Figure 6:**
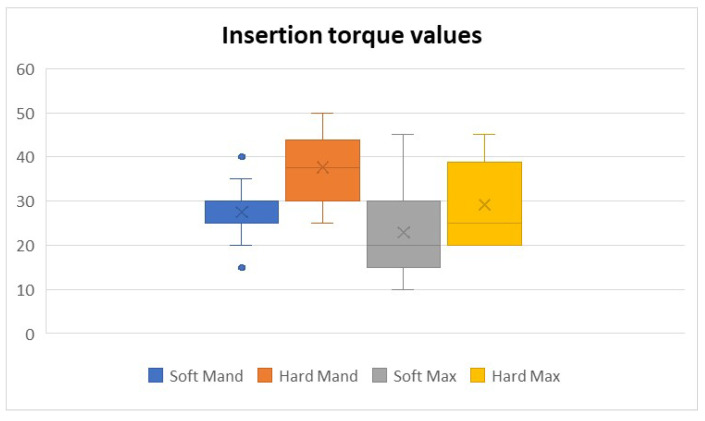
Insertion torque values from the 4 different types of bone areas.

Although the individual anatomy of each individual determines variations in bone density both between samples and within the same sample, the torque values required to insert the implants did not vary greatly, especially in the soft maxillary area (Figure 6).

Analyzing all the insertion toque values, we noticed that for the Hard Mand, the highest torque values were achieved, whereas the Soft Max manifested the lowest values. From the three different types of implant types, the L implant type had the most similar values in all regions.

The division of morphological areas into Soft and Hard areas was carried out mainly to facilitate statistical analysis. During clinical interventions, we often noticed significant differences in density, which also translated into significant differences in the torque required for insertion within the same morphological area.

**Figure 7:**
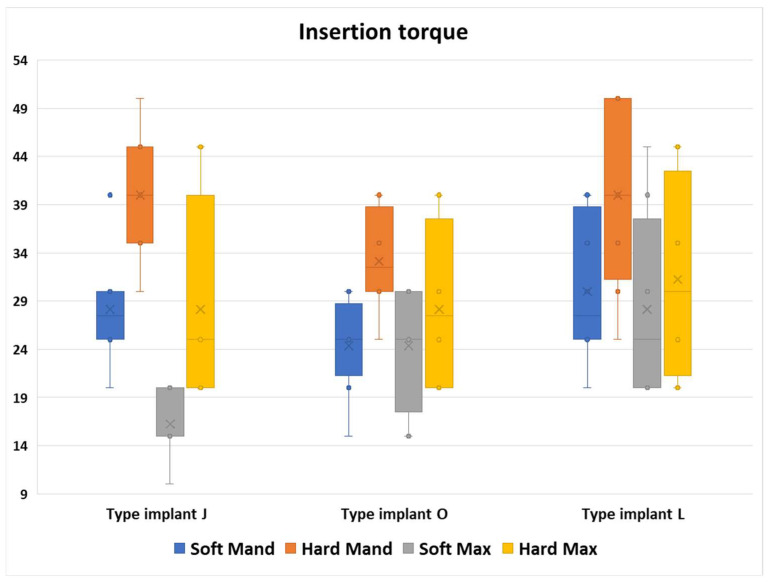
Differences in the insertion torque values from the 3 different types on implants in all areas.

Insertion in areas considered to have high density required the use of torques with high values but also with large differences between mini-implants inserted in the same area due to the high morphological variability, especially in the maxilla but also in the mandible for all three types of mini-implants (Figure 7).

For each anatomical region, a Kruskal–Wallis test was applied to evaluate potential differences in torque values among groups with different implant types. Visual inspection of boxplots indicated that the torque value distributions were comparable across all groups. In both mandibular regions and in the hard maxillary region, the median torque values did not differ significantly among the three implant types (*p* > 0.05) (Table 3).

In the soft maxillary region, however, median torque values varied significantly between implant types, χ^2^ (2) = 9.353, *p* = 0.009. To further explore these differences, pairwise comparisons were conducted using Dunn’s (1964) test, applying a Bonferroni correction for multiple comparisons. Statistical significance was set at *p* < 0.016667, and adjusted *p*-values are reported. The analysis showed a significant difference between implant type J and implant type L (*p* = 0.004). No other comparisons reached statistical significance, although the difference between types J and O approached the adjusted significance threshold (*p* = 0.055) (Table 3).

This graph confirms the results from the previous figure that the highest torque values were required in the Hard Mand region. Moreover, a wide variety of insertion values was obtained in the L implant types regardless of the bone area.

Regarding the median values, we can observe that the toque values required for the J implants were the most dependent on the morphological area.

From all bone areas, we noticed that in the Soft Max area, we registered the greatest variety of the median values (*p* = 0.009, Table 3).

**Figure 8:**
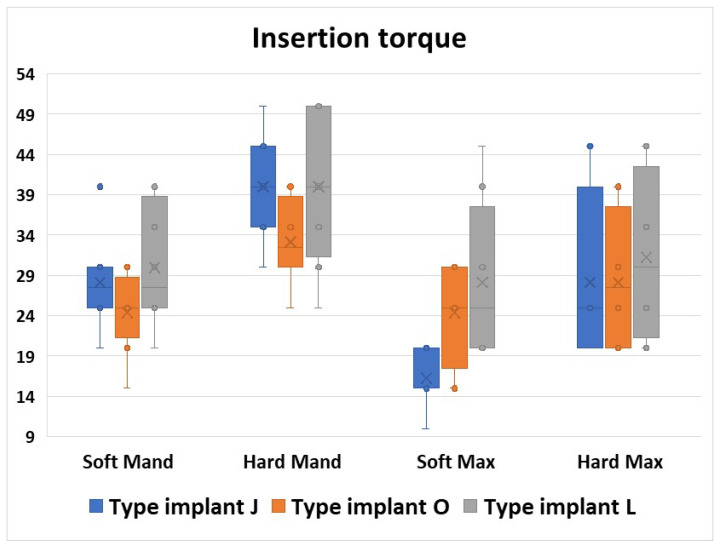
All the insertion torque values from the 3 implant types organized in regions.

From all the regions in the Hard Max area, we noticed similar values and also a similar range in the torque values for all three implants used (Figure 8).

The J type implants showed big differences in the torque insertion values. The values varied depending on the region, but there were also differences in the same areas and also between the two insertions.

From all morphological areas, the lowest values were obtained in the Soft Max area in both insertion moments.

**Figure 9:**
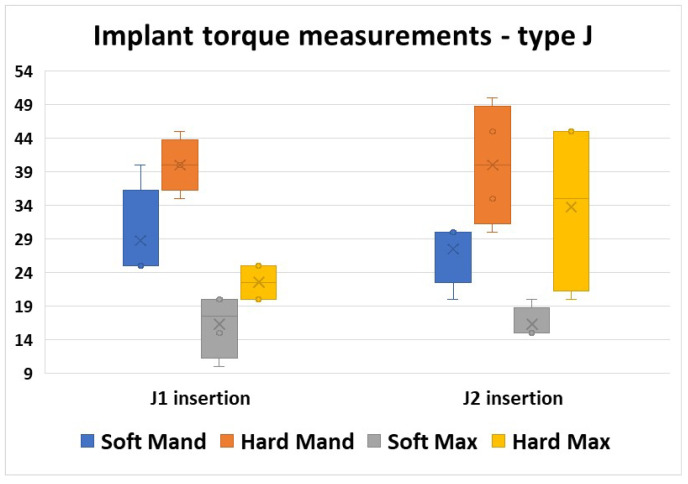
Torque values obtained in all regions for the two insertions of the J type implants.

From all the areas, in the Hard Max area, we obtained the most different values between the two insertion moments, both regarding the mean values, but also the value intervals (Figure 9).

For all four areas, a Mann–Whitney U test was run to determine if there were differences in torque values between the two insertion moments. Distributions of the torque values for J1 insertion and J2 insertion were similar, as assessed by visual inspection. Median torque values for J1 and J2 insertion moments were not statistically significantly different (*p* > 0.05, Table 4).

The O implants showed rather homogeneous values in the four regions, but also between the two insertions.

The intervals of values obtained were not very large, and rather condensed, but they were similar both between the two insertion moments and also in all bone areas.

**Figure 10:**
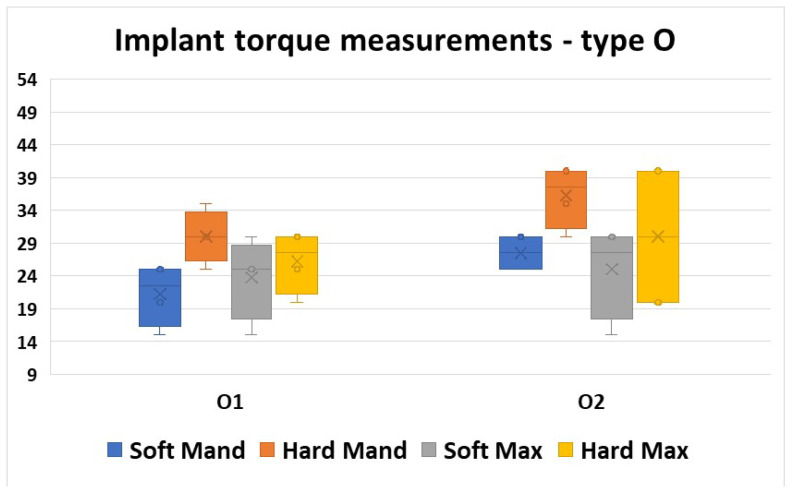
Torque values obtained in all four regions for the two insertions of the O type implants.

The mean values had also similar levels in all areas, and also in the two insertion moments (Figure 10).

For all four areas, a Mann–Whitney U test was run to determine if there were differences in torque values between the two insertion moments. Distributions of the torque values for O1 insertion and O2 insertion were similar, as assessed by visual inspection. Median torque values for O1 and O2 insertion moments were not statistically significantly different (*p* > 0.05, Table 5).

**Figure 11:**
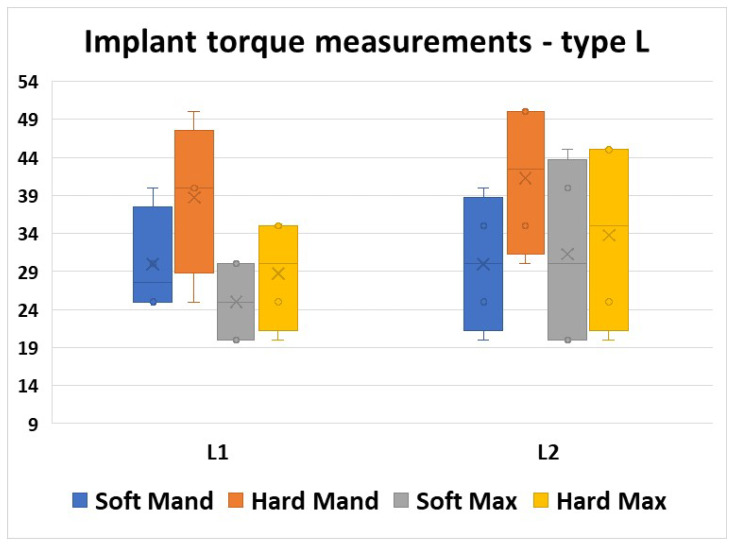
Torque values obtained using the L type implants.

Analyzing the values obtained using the L type implants, we noticed good homogeneity, but with a rather larger band of values compared to the O type implants, where the values were more condensed. The mean values were similar in all morphological areas and also in the two insertion moments (Figure 11).

For all four areas, a Mann–Whitney U test was run to determine if there were differences in torque values between the two insertion moments. Distributions of the torque values for L1 insertion and L2 insertion were similar, as assessed by visual inspection. Median torque values for L1 and L2 insertion moments were not statistically significantly different (*p* > 0.05, Table 6).

From this graph, we can observe the difference in values for the J implants in the four regions compared with the other two implant types. In this implant group, the torque values obtained varied greatly regarding the bone area, with the lowest values obtained in the Soft Max zone, and the highest values in the Hard Mand area.

**Figure 12:**
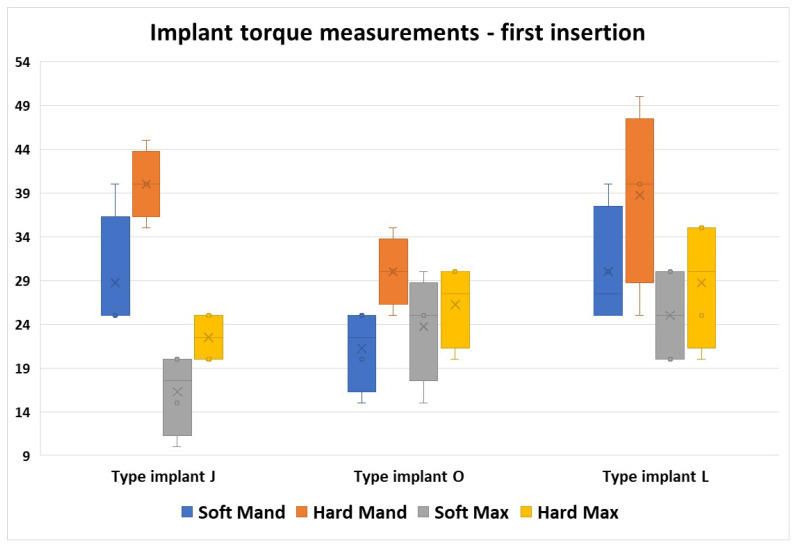
Differences between the 3 implant types in the four regions but only for the first insertions.

From all the implant types, we noticed that the L implant type required a wide variety of torque values, but the interval values obtained were similar between all bone areas (Figure 12).

This figure shows us a larger band of the values, with rather lower differences between the three different implant types compared to the first insertion. However, the larger differences were found in the J implant type.

**Figure 13:**
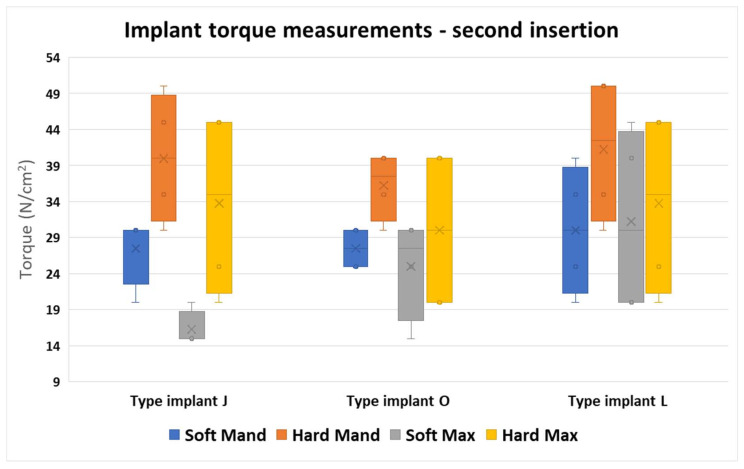
Differences between the torque values of the second insertion of the 3 implant types in the four regions.

From all the values obtained, we noticed that the J implants manifested the greatest variety of torque values in all areas. The O implants had the most similar values, and the most condensed intervals in all areas. The L implant types had the widest range of values; however, the intervals were similar between the bone areas (Figure 13).

**Figure 14:**
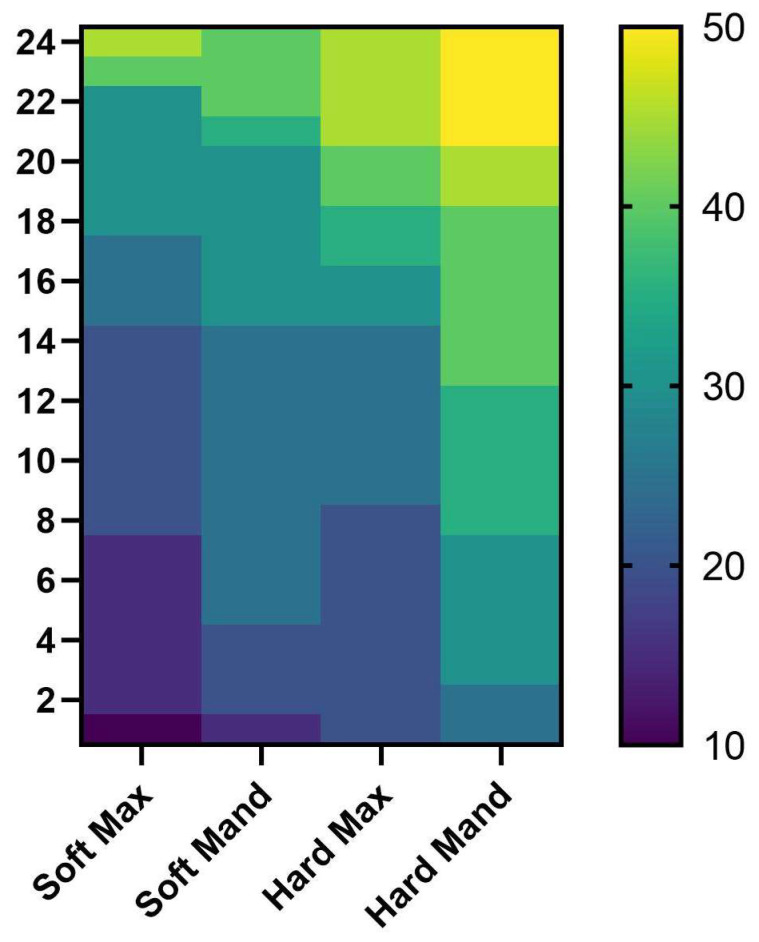
Heat map of all the torque values from all regions.

Analyzing the heat map, where we arranged the bone areas depending on the bone hardness, starting from the Soft Max area which was the softest area, and with the last area which had the highest torque value, we can clearly see that the torque values increased smoothly as we moved to harder bone areas, with the lowest torque values registered in the Soft Max area, and the highest values registered in the Hard Mand area(Figure 14).

## 4. Discussion

In this study, we evaluated the insertion torque values of mini-implants in different morphological areas for three commercial systems because the insertion torque value is directly correlated with their mechanical performance.

For orthodontic anchorage preparation, the use of mini-screws in clinical patients has become widespread in orthodontics. Currently, available orthodontic mini-implants come in a variety of diameters, lengths, body designs, and thread shapes and they can be fabricated from different alloys [7,8]. The tip design of mini-implants also varies; self-drilling types have a cutting tip, allowing direct insertion, while self-tapping types feature a non-cutting tip and require a pilot hole. In a study by Sabley, steel and titanium screws were compared when inserted at different angles in the upper jaw. The results showed no significant differences between the two materials, indicating that both are equally suitable for use in orthodontic practice [9,10].

The stability of orthodontic mini-implants can be affected by the following factors: design, placement procedures, and the quantity and quality of bone relative to the host factor [11].

Multiple studies have explored how variations in mini-implant design influence their primary stability [10]. Heo et al. investigated the placement of tapered mini-implants into thick cortical bone using angled pre-drilling and reported improved primary stability, which they attributed to higher maximum insertion torque and similar total insertion energy values [12]. Suzuki et al. found that self-drilling mini-screws provide significantly greater primary stability compared with pre-drilled implants. They also exhibited reduced osseointegration, which facilitates safer removal and decreases the likelihood of fracture [13]. Similarly, conical mini-implants have shown enhanced stability due to their closer adaptation to bone surfaces [14,15]. However, Siegele and Soltesz noted that conical designs may induce higher cortical bone stress than cylindrical ones [10,16].

In preclinical research, large animal models offer notable benefits over smaller species for investigating human diseases and evaluating new therapies. Among these, pigs have gained popularity in medical studies for scientific, economic, and ethical reasons. Their oral and maxillofacial anatomy, development, physiology, and disease patterns closely resemble those of humans, making them a valuable in vivo model for bone regeneration studies [17].

Animal models are important for understanding the biomechanical mechanisms of orthodontic mini-implants on bone substrate [18]. In our study, to avoid animal sacrifice for ethical reasons as noted by other studies [19], we used bone segments from animals already sacrificed for food purposes.

In our study, we have selected pork mandibles, as some studies in the past have shown that pork bones have the thickness of cortical bone similar to the one of human jaw. Pork ribs specifically have a lower but more homogeneous density [20].

As pig models have been widely utilized to investigate orthodontic mini-implant placement, they also offer important data related to torque, primary stability, and mechanical behavior under various anatomical and insertion conditions. These studies highlight the relevance of porcine bone for simulating human bone properties, especially when analyzing factors such as insertion angle, bone density, and stress distribution [19,21,22]. In our study, we selected four mini-implant placement areas with different morphological characteristics in terms of density. This is important in current clinical practice because bone density directly affects the insertion tension of the mini-implant [23], but also its integration [24] and even its surface changes [25]. This morphological variability justifies and even requires a judicious preliminary preparation of each case with an appropriate clinical and radiological evaluation including complex imaging examinations such as CBCT [26,27].

A more aggressive morphology of the threads allows for easier insertion in areas with increased density [28]. In our study, samples from the Osstem system required the lowest insertion torques in the study groups corresponding to areas with increased density in both the maxilla and mandible.

The palatine arch is an area with high bone density but with particular clinical importance for cases where an expander is applied [29]. Miniscrew-assisted rapid palatal expansion is now frequently used to avoid more traumatic surgical procedures [30]. Obtaining a clinically useful torque, but without deforming the mini-implant is extremely important.

Insertion torque has been found to correlate with screw position, primarily due to the mandible’s thicker cortical bone and superior bone quality compared to the maxilla. While prosthetic implant stability is more affected by the implant’s length and diameter within the alveolar bone than by cortical bone thickness, this study demonstrated a significant relationship between placement torque and cortical bone density [11].

The results obtained in this study align and complement the existing literature on the mechanical behavior of mini-implants. The findings of our study, in comparison with previous research, confirm the significant influence of torque on the mechanical performance of mini-implants. The observed increase in ultimate shear stress and attachment stiffness with higher values of torque align with previous studies that highlighted the importance of appropriate torque introduction to achieve better stability and resistance to failure.

Mini-implants can be manufactured from various alloys, and their tip design also differs. Those with a cutting tip are classified as self-drilling, allowing direct insertion, while those with a non-cutting tip are considered self-tapping and require a pilot hole to be prepared at the surgical site. The properties of orthodontic mini-implants, including their dimensions, designs, and placement protocols, have become increasingly diverse to enhance clinical efficiency [12,28]. While several studies have investigated how factors such as implant size, insertion angle, cortical bone thickness, and bone density influence success rates and reduce failure, relatively few have examined the outcomes of reusing mini-implants after prior use [31]. During the placement of mini-screws, a certain amount of insertion torque is needed in order to achieve good primary stability. However, an excessive torque can lead to fractures in the cortical bone and bone resorption, and hence to failure of the mini-screw [32].

The insertion value is not a fixed value for all patients, as it is influenced by the morphological variability of the bone, which presents different degrees of bone density. Our study confirms what we encounter daily in clinical practice, namely the need for higher torque in areas considered HARD, as well as significant inter-individual morphological variability. Other studies have also highlighted the need for higher torque in areas with increased bone density, which may serve as a marker not only for the anatomical structures encountered [33,34]. Motoyoshi et al. [35] suggested that an ideal insertion torque for higher success rates should range between 5 and 10 Ncm. However, a systematic review [2] found no supporting evidence for this recommendation and concluded that no specific insertion torque value can be reliably endorsed as more effective.

In our study, we employed the self-drilling technique without pre-drilling. Heidemann et al. [36] reported that when pre-drilling is used, the pilot hole should not exceed 80% of the screw’s diameter to ensure optimal primary stability and maintain ideal insertion torque [37]. The risk of fracture of orthodontic mini-implants depends on other variables, such as the diameter, length, geometric design, and insertion angle, in addition to the type of alloy chosen [6,38,39,40]. A smaller diameter leads to a decrease in the structural strength of the mini-implant, making it more susceptible to fracture and deflection [41,42].

Throughout the years, studies have shown that the permanence of the mini-implants, as well as the secondary stability, are heavily influenced if primary stability is achieved. The primary stability will allow the mini-implants to receive the necessary loads and the chances of successful treatment are greater [43].

Unlike conventional endosseous implants, which require a healing period for bone integration, mini-implants rely primarily on mechanical retention to achieve primary stability. Therefore, in vitro studies on mini-screws should aim to more accurately simulate their response to immediate loading following placement [44].

Studies have shown that the diameter of the mini-screw has a major influence on the mini-implant stability. Increasing the mini-screw extrabony head length can cause an increase in the stress forces in the bone and therefore can compromise the stability. When placing mini-implants in the palatine alveolar areas, longer heads are inevitable because of increased soft tissue thickness. In these situations, a mini-screw with a larger diameter is recommended to enhance stability.

Other factors that are considered to affect the quality of the mini-implant stability are the cortical bone thickness and cancellous bone quality [45,46]. It has been proven that higher placement torque values can overheat the bone in vivo and result in screw failure [47,48]. Clinically, failures at higher torque values might also be due to higher masticatory forces and limited access for oral hygiene, or provide a more difficult location for screw placement [49].

By comparing the values obtained at the two insertion moments, we observed a slight increase in the average torque required for insertion. However, the main characteristic was the greater variability in the insertion torque, expressed by a wider range of values across all implant types and all morphological zones, particularly in the HARD zones. Ozkan also concluded in 2022, after a four-week study on human subjects, that the differences during second use are insignificant when an effective cleaning protocol is applied [50]. Because of increased force of insertion or higher torque values needed when placing mini-implants, there is also a high risk of microfractures in those bone areas. Clinicians have reported a wide range of mini screw success rates, but a protocol for the screw placement has yet to be standardized. Although many factors play a role in the success or failure of mini-screws, the most essential factor remains the placement torque value [51].

In our study, we analyzed a torque measurement provided by the physiodispenser used. In the literature, there are a number of concerns related to the validity of the measurements that we can obtain using common dental torque instruments [52,53,54]. These aspects require a more in-depth evaluation that must be taken into account in future studies.

In vitro testing allows for better standardization of results while in vivo testing allows us to have a closer picture of clinical reality. Although we used bone segments from already sacrificed animals, there was quite a large variability in the bone morphology encountered. The limitations of this study are also given by the relatively small number of available bone segments that were used. Even though in order to limit animal sacrifice, the samples were obtained from a local butcher shop, their degree of variability was quite high, which meant that the number of bone segments with a similar anatomy was still reduced. For future studies, we aim to identify other sources from which we can choose more bone samples in order to have results with a greater statistical value.

## 5. Conclusions

Although there is no unanimously accepted value of the mini-implant insertion torque and it is widely accepted that a too high value may affect the success of the insertion, the results of this study showed that the torque required to insert the mini-implants differs depending on the anatomical area and the mini-implant system.

Insertion in areas considered to have high density requires the use of torques with high values but also with large differences between mini-implants inserted in the same area due to the high morphological variability, especially in the maxilla but also in the mandible for all three types of mini-implants.

Moreover, as a practitioner, if one needs to reuse a mini-implant, one must expect a greater value and especially a larger variability of the necessary torque, depending on the morphological area.

The morphological variability of the bone substrate causes the torque value to vary even within the same type of tissue, making it difficult to identify a standard value for a specific type of mini-implant system. The results of this study highlight the need for continued research to analyze the ideal insertion torque of different types of mini-implants depending on the insertion area, in order to achieve clinical success of mini-implants.

## Figures and Tables

**Table 1:** List of the tested screws and their characteristics offered by their producers.

Feature	Jeil S20-JD-010	Osstem OSSH1810	Leone 003-2009-10
Diameter	2.0 mm	1.8 mm	2.0 mm
Length	10 mm	10 mm	9 mm
Thread Design	Fine-pitch threads with self-tapping flutes; cylindrical-tapered transition	Corkscrew-style dual-thread (wider lower + finer upper)	Cylindrical self-tapping coil; thread ~1.75 mm
Implant Tip Geometry	Sharp, conical self-drilling tip with cutting edge and flutes	Sharp, self-drilling apical tip; likely conical	Sharp fluted drill-tip, 2 mm long; self-drilling
Material	Ti-6Al-4V (Grade 5 Titanium)	Ti-6Al-4V (Grade 5 Titanium)	Ti-6Al-4V (Grade 5 Titanium)
Insertion Technique	Self-drilling or predrilling (optional, for dense bone)	Self-drilling (predrilling may be needed in dense bone	Self-drilling (predrilling optional in dense bone)

**Table 2:** Torque values distributed by implant types and zones.

Zone	Parameter	Torque Values (N/cm^2^)		
		Implant Type J	Implant Type O	Implant Type L
Soft Mand	Minimum	20	15	20
	Maximum	40	30	40
	Median	27.50	25.00	27.50
	Variance	35.268	24.554	57.143
	Confidence interval—lower bound	23.16	20.23	23.68
	Confidence interval—upper bound	33.09	28.52	36.32
Hard Mand	Minimum	30	25	25
	Maximum	50	40	50
	Median	40.00	32.50	40.00
	Variance	42.857	28.125	92.857
	Confidence interval—lower bound	34.53	28.69	31.94
	Confidence interval—upper bound	45.47	37.56	48.06
Soft Max	Minimum	10	15	20
	Maximum	20	30	45
	Median	15.00	25.00	25.00
	Variance	12.500	38.839	99.554
	Confidence interval—lower bound	13.29	19.16	19.78
	Confidence interval—upper bound	19.21	29.59	36.47
Hard Max	Minimum	20	20	20
	Maximum	45	40	45
	Median	25.00	27.50	30.00
	Variance	113.839	70.982	105.357
	Confidence interval—lower bound	19.21	21.08	22.67
	Confidence interval—upper bound	37.04	35.17	39.83

**Table 3:** Torque median values, expressed as N/cm^2^.

Area	Implant Type (Median Values)			*p* *
	Type J	Type O	Type L	
Soft Mand	27.5	25	27.5	0.318
Hard Mand	40	32.5	40	0.127
Soft Max	15	25	25	0.009 ^#^
Hard Max	25	27.5	30	0.749

* Kruskal–Wallis H test. ^#^ Statistically significant.

**Table 4:** Torque median values expressed as N/cm^2^, for the implant type J, divided by insertion moment.

Area	Implant Type J		*p* *
	J1 Insertion	J2 Insertion	
Soft Mand	25	30	1.000
Hard Mand	40	40	1.000
Soft Max	17.5	15	0.886
Hard Max	22.5	35	0.343

* Mann–Whitney U test.

**Table 5:** Torque median values expressed as N/cm^2^, for the implant type O, divided by insertion moment.

Area	Implant Type O		*p* *
	O1 Insertion	O2 Insertion	
Soft Mand	22.5	27.5	0.114
Hard Mand	30	37.5	0.114
Soft Max	25	27.5	0.686
Hard Max	27.5	30	1.000

* Mann–Whitney U test.

**Table 6:** Torque median values expressed as N/cm^2^, for the implant type L, divided by insertion moment.

Area	Implant Type L		*p* *
	L1 Insertion	L2 Insertion	
Soft Mand	27.5	30	0.886
Hard Mand	40	42.5	1.000
Soft Max	25	30	0.686
Hard Max	30	35	0.686

* Mann–Whitney U test.

## Data Availability

The original contributions presented in the study are included in the article, further inquiries can be directed to the corresponding author.
